# ﻿*Euonymusaquifolium* (Celastraceae): Rediscovered in flowering with respect to its taxonomy, nomenclature, and rarity

**DOI:** 10.3897/phytokeys.201.86180

**Published:** 2022-07-05

**Authors:** Jun Hu, Jun-Yi Zhang, Ding-Xiang Yu, Hong Jiang, Bo Xu, Qing Liu, Hai He

**Affiliations:** 1 CAS Key Laboratory of Mountain Ecological Restoration and Bioresource Utilization & Ecological Restoration and Biodiversity Conservation Key Laboratory of Sichuan Province, Chengdu Institute of Biology, Chinese Academy of Sciences, Chengdu 610041, China Chengdu Institute of Biology, Chinese Academy of Sciences ChengDu China; 2 College of Life Sciences, Chongqing Normal University, Chongqing 401331, China University of Chinese Academy of Sciences Beijing China; 3 Ecological Restoration and Conservation of Forests and Wetlands Key Laboratory of Sichuan Province, Sichuan Academy of Forestry, Chengdu 610081, China Chongqing Normal University Chongqing China; 4 University of Chinese Academy of Sciences, Beijing 100049, China Ecological Restoration and Conservation of Forests and Wetlands Key Laboratory of Sichuan Province, Sichuan Academy of Forestry ChengDu China

**Keywords:** endemic species, *Euonymusaquifolium*, flora of China, floral character, *Glyptopetalum*, nomenclature, taxonomy

## Abstract

A small population of *Euonymusaquifolium* (Celastraceae) with flowering plants was discovered more than 100 kilometers away from its type locality. The incomplete original description of this species is supplemented here with floral materials since it is known from only two gatherings of fruiting specimens. Its 5-merous flowers and two ovules per locule support its position in *Euonymus*, and this inference is further reinforced by phylogenetic analysis based on the nuclear internal transcribed spacer (ITS) of rDNA. The specific epithet has long been treated as “*aquifolius*” to agree with the generic gender of *Euonymus*. But after examination of the protologues of this and other related species described simultaneously by the same authors, as well as their handwritten annotations attached on the type specimens, we conclude that the epithet *aquifolium* was used as a noun and it should be retained unchanged. Despite this newly discovered population some 100 kilometers away from its type locality, this species is still assessed as Critical Endangered (CR) according to the IUCN Red List Categories and Criteria.

## ﻿Introduction

*Euonymusaquifolium* Loes. & Rehder in Sargent (1913) was described based on a single gathering^1^ collected by E. H. Wilson in 1908 from West Sichuan. It was incompletely known up until now since the three duplicates of the type gathering (*E. H. Wilson 1366*) were branches in fruit, and the other documented gathering (*P. N. Qin et al. 104* collected in 1929) was reported as bearing neither flowers nor leaves (Cheng et al. 1999). The account of this species in the Chinese flora (Cheng et al. 1999; Ma et al. 2008) and Sichuan flora (Chang 1988) was based only on these two gatherings. Being endemic to Sichuan, and without any information on its living population in the wild, it was assessed as a critically endangered species (CR) in the China Biodiversity Red List (Anonymous 2013). However, with its distinctive leaf shape and texture, it would be unusual to be neglected by more recent botanists during their fieldwork in and around the type locality.

Based on uncertain observations on the fruiting materials, the type specimens (from the description in the protologue) and *P. N. Qin et al. 104*, Cheng & Ma in Cheng et al. (1999) transferred this species to the genus *Glyptopetalum*Thwaites (1856). The name *G.aquifolium* (Loes. & Rehder) C.Y.Cheng & Q.S.Ma in Cheng et al. (1999) was followed by Ma et al. (2008) and Anonymous (2013). *Glyptopetalum* is morphologically very close to *Euonymus*Linnaeus (1753), from which it was defined to differ in the exclusively 4-merous flower, single pendulous ovule per locule of ovary, and seed with branched raphe (Thwaites 1856; Ding Hou 1963; Simmons 2004; Ma et al. 2008). Since its publication, some authors either did not mention *Glyptopetalum* (e.g., Kurz 1877; Loesener 1901–1902), or at most treated it as a subdivision of *Euonymus* (e.g., Kurz 1875; Baillon 1877), while others adopted it as a distinct genus and added newly described species or transferring previously described species, mostly from *Euonymus* (e.g., Prain 1891; Ding Hou 1963; Meng et al. 2011). *Glyptopetalum* is a less speciose genus with ca. 20 species than the ~ 130 species of *Euonymus* (Simmons 2004; Ma et al. 2008; Simmons et al. 2012). Subsequent phylogenetic studies revealed unequivocally that the 2–3 sampled *Glyptopetalum* taxa were a clade nested within *Euonymus* (Simmons et al. 2012; Li et al. 2014), such that *Glyptopetalum* should be included within a broadly defined *Euonymus* (Li et al. 2014).

In August 2021, during a field trip of the Second Tibetan Plateau Scientific Expedition in Jiulong County, Sichuan, Southwest China, an area located along the southeastern slope of Mt. Gongga (Minya Konka, the main peak of Hengduan Mountain), a small-sized population of ca. 15 individuals of *Euonymusaquifolium* was unexpectedly encountered by Jun Hu (the first author of this article) and his team members. Some plants were in flower, and the 5-merous flowers instantly reject its identity as a member of *Glyptopetalum*. To better understand this species, its morphological description was thereafter expanded with the observation of living plants and dissection of floral parts. To test whether its generic position inferred using floral characters correspond to the molecular data, a phylogenetic analysis was conducted by incorporating data from published studies with the addition of samples of this species and its morphologically closest species, *Glyptopetalumilicifolium* (Franchet 1886) C.Y.Cheng & Q.S.Ma in Cheng et al. (1999), which was collected in 2021 by Chong-Bo Ma & Dong-Liang Lin (*YDYC137* at CDBI) from Xichang, Southwest Sichuan.

## ﻿Materials and methods

### ﻿Morphological description

*Euonymusaquifolium* was observed as living individuals in the field, and dried herbarium specimens were observed in laboratory, where morphological characters were measured using ImageJ v1.53 k (Schneider et al. 2012). Our description follows the terminology used by Harris and Harris (2001). Voucher specimens were deposited at CDBI (acronym of herbarium follows Thiers 2022).

### ﻿DNA extraction, amplification and sequencing

Apart from the newly generated data of *Euonymusaquifolium* and *Glyptopetalumilicifolium* in this study, all of the other sequences of the 62 samples representing 51 species in molecular phylogenetic analysis were retrieved from GenBank. The accessions are listed in Appendix I. Total DNA was extracted exclusively from silica-gel dried leaves using a Plant DNA Isolation Kit (Cat.No.DE-06111). The same primers and outgroups were used as the phylogenetic analysis of *Euonymus* by Li et al. (2014). The nuclear internal transcribed spacer (ITS) was amplified by polymerase chain reaction (PCR). All DNA samples were sent to TSINGKE Biotech Co. Ltd (Chengdu, China) for sequencing and then deposited to GenBank under the accession number OK172405 for *Euonymusaquifolium* and OM985812 for *Glyptopetalumilicifolius* (Appendix I).

### ﻿Phylogenetic analyses

All sequences were processed with Sequencher v4.1.4 (Gene Codes, Ann Arbor, Michigan, USA), and aligned by using MAFFT v7.475 (Katoh and Standley 2013) with default parameters. Maximum likelihood (ML) and Bayesian inference (BI) methods were applied to infer the gene tree. jModeltest 2.1.6 (Posada 2008) identified GTR+I+G as the best model which selected using the corrected Akaike Information Criterion (AICc). BI analysis was conducted using MrBayes 3.2.7a (Ronquist and Huelsenbeck 2003) with two parallel runs (10 million generations). The first 25% percent of trees from all runs were discarded as burn-in. ML analysis was performed using IQ-TREE v.1.4.241 (Nguyen et al. 2014) with branch support estimated using 2,000 replicates of ultrafast bootstrapping algorithm (UFboot) (Minh et al. 2013).

## ﻿Results and discussion

**Figure 1.**
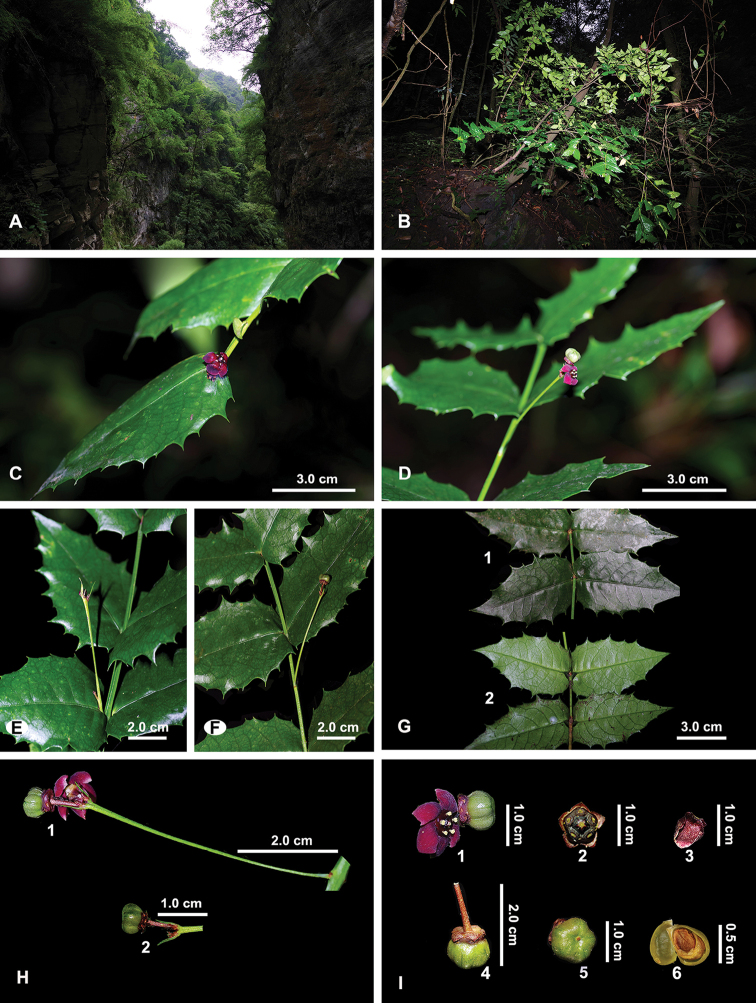
*Euonymusaquifolium* Loes. & Rehder **A** habitat **B** habit on cliff **C** branch with a flower **D** a flower and an immature fruit **E** axillary inflorescence **F** extra-axillary inflorescence **G** leaves in adaxial (G1) and abaxial views (G2) **H** an inflorescence showing peduncle (H1) and pedicel (H2) **I** close-up of a flower and an immature fruit (I1), showing disk and calyx (I2), a detached petal (I3), an immature fruit in side view (I4) and front view (I5), and two immature seeds in a fertile locule with the lower right one covered by aril.

**Figure 2.**
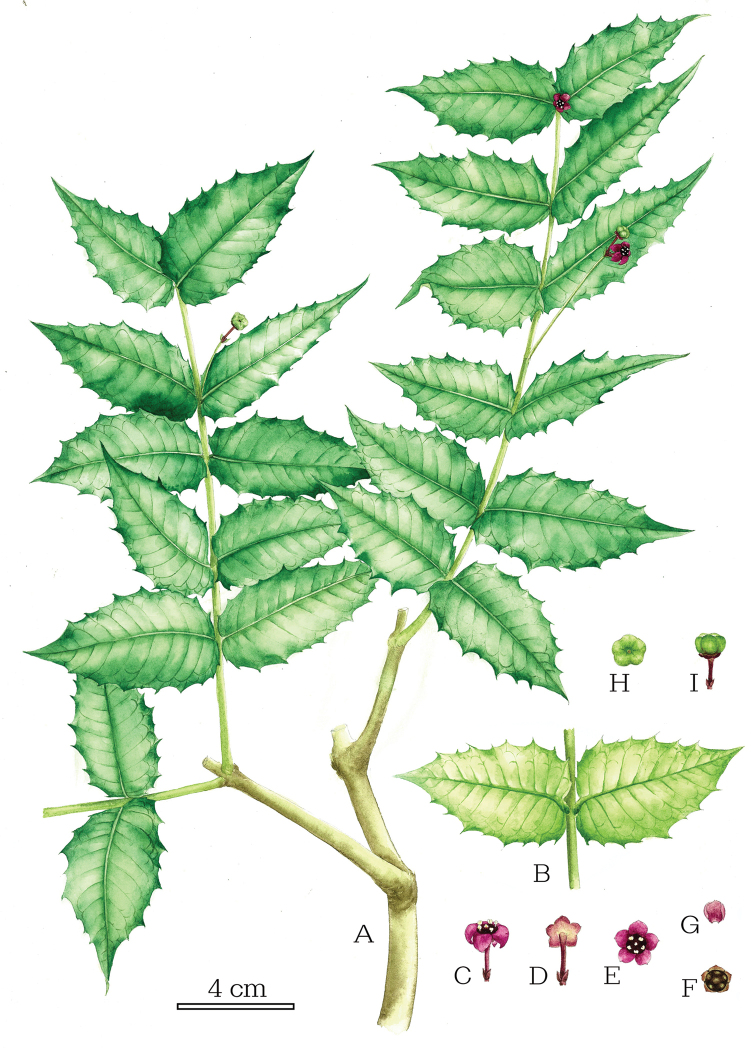
*Euonymusaquifolium* Loes. & Rehder **A** flowering and fruiting branches **B** leaves in abaxial view **C** a flower in side view **D** a flower in rear view showing abaxial calyx, attached pedicel and a pair of bracteoles at its base **E** a flower in front view **F** a flower with petals removed showing disk **G** a detached petal **H** an immature fruit in front view **I** an immature fruit in side view [Drawn by Cong-Ying Li from live specimens *J. Hu et al. hujun20210810B01*].

From the field observation, the general morphology of *Euonymusaquifolium* (Figs 1, 2) agreed well with the description in Sargent (1913) protologue. The fruits were mostly yellowish green, glabrous rather than “squarrulose maculate” as documented in the Chinese floras (Cheng et al. 1999; Ma et al. 2008), which might be an error caused by confusing the fruiting characters of *Glyptopetalumilicifolium* (from our observation of the gathering *YDYC137* and referring to Franchet 1886; Cheng et al. 1999; Ma et al. 2008). The flowers were clearly 5-merous, with five sepals, petals, and stamens, and 5-locules in well-developed ovaries. There were 2 ovules at the center of the axis of placenta, and usually only one ovule developing into seed, which might explain the description in the protologue as “1–2 seeds” per locule. We observed that one or more locules generally aborted in fruits, which makes some of the fruits in the pressed specimens appear 4-loculed, or even 2-loculed. For the immature fruits in this newly discovered population, no branched raphe on the seeds was observed. Detailed morphology with special attention to the supplemented floral characters is provided in the following description.

**Figure 3.**
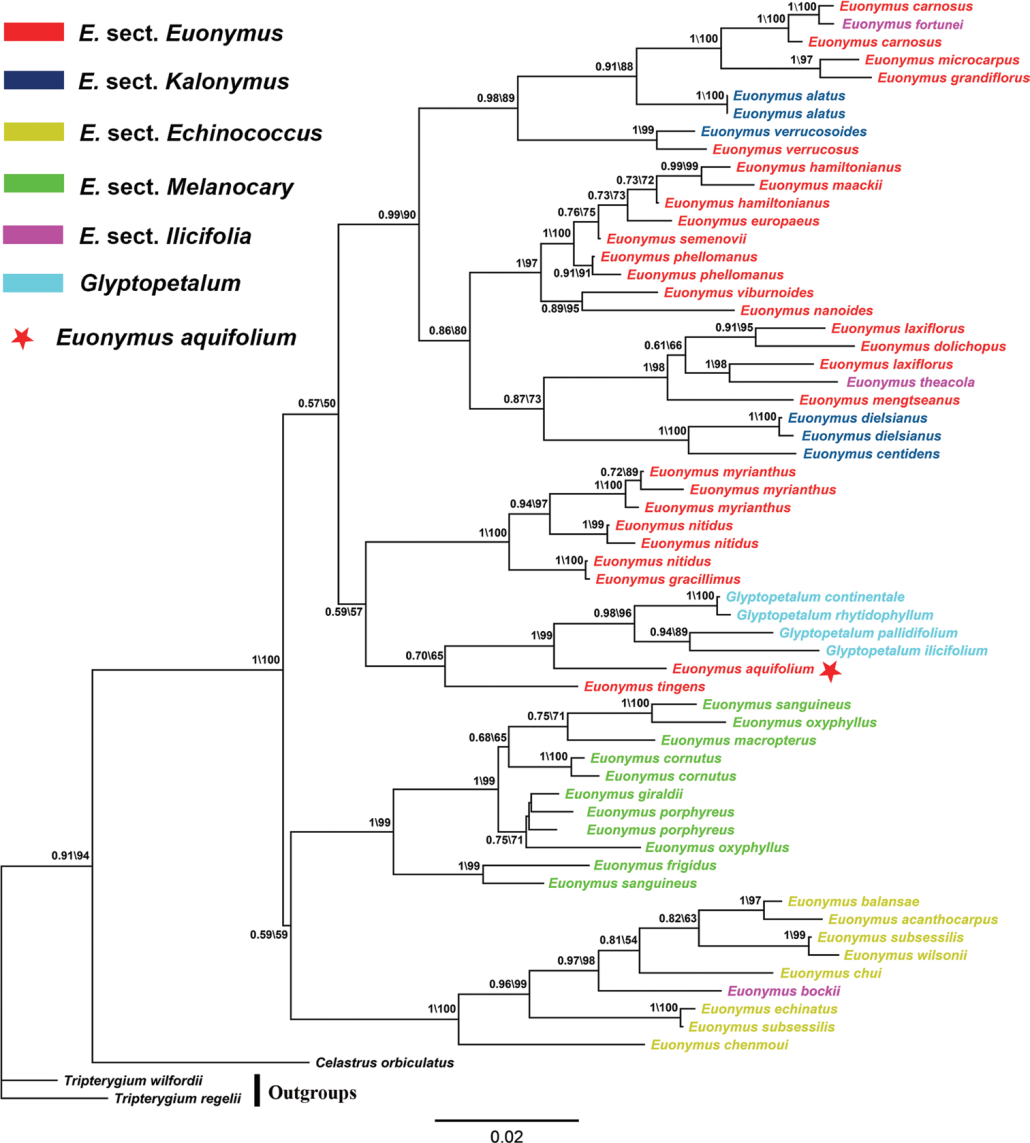
Bayesian and ML-based phylogenetic tree inferred from nuclear internal transcribed spacer (ITS) of the 51 sampled taxa identified as species of *Euonymus* and/or *Glyptopetalum*. Values above branches are Bayesian posterior probabilities (> 0.5) / maximum likelihood bootstrap percentages (> 50). Colors of terminal nodes correspond to the five sections of *Euonymus* defined in *Flora of China* (Ma et al. 2008) and the genus *Glyptopetalum*.

With the addition of newly generated *Euonymusaquifolium* and *Glyptopetalumilicifolium* sequences, the molecular phylogenetic tree revealed overall similar resolution (BI / ML = 1 / 100; Fig. 3) of *Euonymus* sensu lato as the previous study by Li et al. (2014). Together with the three samples identified as *Glyptopetalum*, i.e., *G.continentale* (Chun and How 1958) C.Y.Cheng & Q.S.Ma in Cheng et al. (1999), *G.rhytidophyllum* (Chun and How 1958) C.Y.Cheng in Cheng et al. (1999), and *G.pallidifolium* (Hayata 1913) Q.R.Liu & S.Y.Meng in Meng et al. (2011), the two newly sampled taxa were resolved in a well-supported clade (1/99) sister to *Euonymustingens* Wall. in Roxburgh (1824). All four sampled *Glyptopetalum* species are nested within *Euonymus*, which supports synonymization of *Glyptopetalum* with *Euonymus* pending further phylogenetic studies including its generic type *Glyptopetalumzeylanicum*Thwaites (1856). Moreover, our inferred tree reinforces the inference from previous studies that delimitations of sections within *Euonymus* remain problematic (Simmons et al. 2012; Li et al. 2014), except for E.sect.Melanocarya (Turczaninow 1858) Nakai (1941).

In summary, the results of both floral morphological observation and molecular analysis support its retention in *Euonymus*.

### ﻿Taxonomic treatment

#### ﻿ Euonymus aquifolium

Taxon classificationPlantaeCelastralesCelastraceae

Loes. & Rehder in Sargent 1913: 484

90F0C2B1-8829-5C90-961F-9AA96C08CDB1

Figs 1 , 2

≡ Glyptopetalumaquifolium (Loes. & Rehder) C.Y.Cheng & Q.S.Ma in Cheng et al. 1999: 93

##### Type.

**Figure 4.**
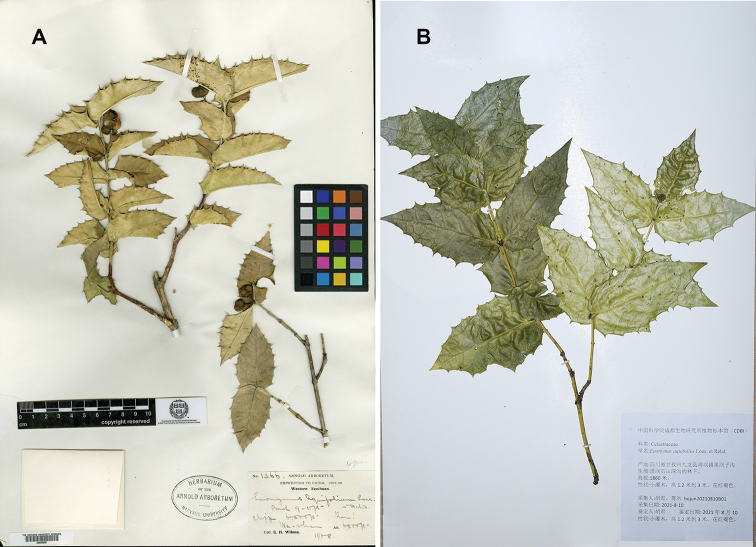
Selected specimens of *Euonymusaquifolium* Loes & Rehder **A** holotype of this species (*E. H. Wilson 1366*, A00049691) **B** a duplicate of *J. Hu et al. hujun20210810B01* (CDBI).

China. Sichuan [Szechuan]: Wa-shan, on cliffs, elev. ca. 2200 m, in fruiting, November 1908, *E. H. Wilson 1366* (holotype A00049691 (Fig. 4A); isotypes K000669647 & US00096036).

##### Description.

Evergreen shrubs, 1–3 m tall, glabrous throughout; young branches firstly 4-angular and green, later turning almost terete and grey-brown. Leaves opposite, leaf blade leathery, oblongly ovate, ovate to orbicularly ovate, 4–7 × 2.5–4.5 cm, uneven on both surfaces, adaxially dark green, abaxially slightly pale green, glossy, margin with clear and irregular large spines, apex acute or short acuminate, base slightly cordate and often marginally clasping the branch, more or less oblique; lateral veins 6–10 pairs, curved and distally ca. 1/4 its length near margin connected with tertiary veins, midrib and lateral veins visibly elevated on both surfaces, transverse veins obscure; subsessile or petiole to 2 mm long. Cymes in leaf axils or extra-axillary, nearly on the adaxial side of branchlet when extra-axillary, with 1 to several (mostly 5–7) flowers; peduncle 1.5–5 cm long; pedicel 0.6–1.2 cm long, usually with two opposite bracteoles at base; bracteoles subulate, 0.4–0.8 cm long, ca. 2 mm wide, persistent. Flowers red-brown, 1–1.5 cm in diameter, 5-merous; calyx 5-lobed to middle, lobes triangular, red-brown, margin with fleshy projections, persistent; petals 5, fleshy, 0.5–0.8 cm long, 0.3–0.6 cm wide, broad-ovate, slightly revolute; disc pentagonal, deep red, fused to ovary; stamens 5 on disk, filaments very short, anthers small and yellow; ovary partly exposed outside disk, reddish brown, style absent, stigma rounded, white with yellowish tinge; ovary 5-locular; ovules 2 per locule. Capsule, subglobose, mostly yellowish green when immature, glabrous, 8–10 mm high, 1.2–1.5 cm in diameter, 5-loculed, sometimes only 4(–2)-loculed owing to infertile of one or more locules, with 2 seeds per locule, or only one seed with another ovule aborted. Seeds brown, oblong, 0.4–0.8 cm long, with orange-yellow aril, more than 1/2 covered by aril.

##### Phenology.

Flowering was observed in August, and it could start earlier; fruiting from August to November.

##### Habitat.

The newly discovered site (elevation ca. 1850 m) is located under the cliff of a small ditch in the Dadu River Basin. The place is located in the so-called ‘Rain Zone of Western China’, where it is commonly rainy most of the year. The habitat is further shady and humid due to the gorge landform (Fig. 1A, B). Based on the records of type specimens, *Euonymusaquifolium* can grow on the cliffs within the evergreen broadleaf forest or evergreen and mixed deciduous broadleaf forest at an elevational range of 1800–2200 m in Dadu River Basin.

##### Additional specimens examined.

China. Sichuan: Jiulong County, Wanba, elev. ca. 1850 m, in flowering, 10 August 2021, *J. Hu et al. hujun20210810B01* (CDBI! NAS! PE!) (Fig. 4B).

##### Nomenclatural note.

The species epithet of *Euonymusaquifolium* had been changed to “*aquifolius*” to agree with the generic gender based on the assumption that this epithet was used as adjectival in form, and this was generally followed (e.g., Chang 1988; Cheng et al. 1999; Ma et al. 2008; IPNI 2022). However, when it was originally proposed, the initial letter of the epithet was capitalized as “*Aquifolium*” (Sargent 1913 ), and that was customary then to indicate the epithet was applied after a proper noun, such as a person or a genus (Clifford and Bostock 2007). *Aquifolium*Miller (1754) is an illegitimate superfluous generic name of *Ilex*Linnaeus (1753), though it could also be used as an adjective (*aquifolius*). Loesener & Rehder also capitalized the epithet when describing other species in the same publication (Sargent 1913 ). For example: *Euonymussargentianus* Loes. & Rehder in Sargent (1913) by naming the epithet as “*Sargentiana*” (named after a person), and *E.oblongifolius* Loes. & Rehder in Sargent (1913) as “*oblongifolia*”. This conclusion is further supported based on a review of the handwriting annotations by those authors on the type material of these taxa, where the species epithets of *E.aquifolium* (*E. H. Wilson 1366*, A) and *E. sargentianus (E. H. Wilson 1187*, A) were written in uppercase, while that of *E.oblongifolius* (*E. H. Wilson 3125*, A) was in lowercase. Therefore, the species epithet of *Euonymusaquifolium* should retain its own gender and termination according to Art. 23.5 of ICN (Turland et al. 2018).

##### Rarity and conservation status.

**Figure 5.**
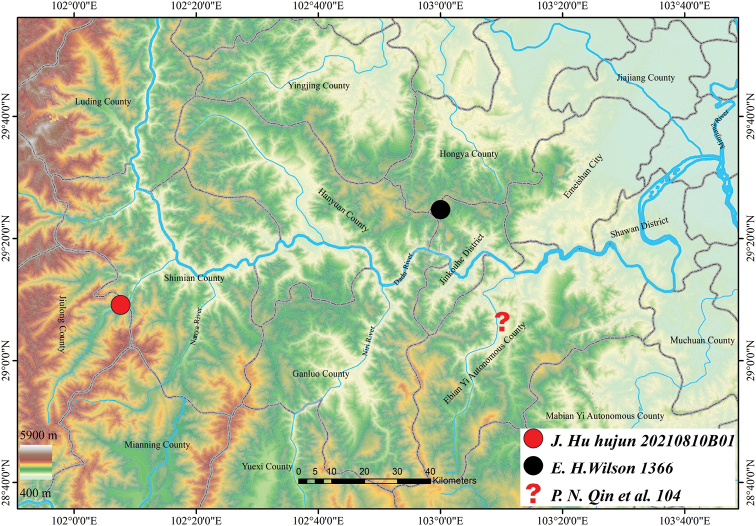
Distribution map of *Euonymusaquifolium* Loes & Rehder based on three collections.

The type material of *Euonymusaquifolium* was collected by E. H. Wilson in November 1908 in Washan, Sichuan. Further geographical information concerning the type locality could not be traced by the related references (such as Wilson 1913, 1929; Yin et al. 2010). Owing to the historical vicissitudes, the picture named as “Wa Shan” in Wilson (1913, 1929) was traced by Yin et al. (2010; Fig. 5) to Jinkouhe District, Leshan City, Sichuan. A few botanists and amateurs (K. P. Yin, pers. comm.) had made attempts to find the living plants of this species around this area without result.

Another gathering (*P. N. Qin et al. 104*) identified as this species is recorded in Cheng et al. (1999) without mention of the herbarium where the specimen(s) were deposited. The collectors were a team of younger volunteers assigned by Tsofu Lu (1893–1952), the director of the then newly established Science Institute of West China, to survey in western Sichuan along the water courses in 1929 (Hou 2012), and most of their collections include duplicates bequeathed to the present Chongqing Natural History Museum (CQNM). An extensive search at CQNM did not identify any material from this gathering (Feng Chen, pers. comm.). It is unimaginable that it lacked leaves as commented in Cheng et al. (1999) since this is an evergreen thick leathery leaved plant. Searches by enthusiastic amateurs previously and Jun Hu’s team recently for living individuals near the locality of this gathering (Fig. 5) were also unsuccessful.

We conclude that *Euonymusaquifolium* is a rare and vegetatively distinctive species, and this rediscovery uncovered the only presently confirmed living individuals more than 110 years after its description, which are distributed more than 100 km westward from its type locality (Fig. 5). Although it is located in difficult-to-access valleys on, and living on a cliff that is unlikely to be destroyed intentionally, natural hazards might still totally damage its habitat. With just ~ 15 individuals, it should still be assessed as Critically Endangered (CR) according to the IUCN (2022) Red List Categories and Criteria.

## Supplementary Material

XML Treatment for Euonymus aquifolium

## ﻿Appendix I

**Table AI.** The GenBank accessions for DNA sequences used in this paper.

Taxon, GenBank accession No. for ITS.
*Euonymus acanthocarpa Franch.*, KF282154; *E.aquifolius* Loes. & Rehder, OK172405; *E.alatus* (Thunb.) Sieb, KF282155, KF282156; *E.balansae* Sprague, KF282157; *E.bochii* Loes. ex Diels, KF282158; *E.carnosus* Hemsl., KF282159, KF282160; *E.centidens* H. Lev., KF282161; *E.chenmoui* W. C. Cheng, KF282162; *E.chuii* Handel-Mazzetti, KF282163; *E.cornutus* Hemsl., KF282164, KF282165; *E.dielsiana* Loes. ex Diels, KF282166, KF282167; *E.dolichopa* Merr. ex J. S. Ma, KF282168; *E.echinatus* Wall., KF282169; *E.europaeus* L., KF282170; *E.frigida* Wall., KF282171; *E.giraldii* Loes. ex Diels, KF282172; *E.gracillimus* Hemsl., KF282173; *E.grandiflora* Wall., KF282174; *E.hamiltonianus* Wall. ex Roxb., KF282175, KF282176; *E.heaeracea* Champ. ex Benth., KF282177; *E.laxiflora* Champ. ex Benth., KF282178, KF282179; *E.maackii* Rupr., KF282180; *E.macroptera* Rupr., KF282181; *E.mengtseanus* (Loes.) Sprague, KF282182; *E.microcarpa* (Oliv. ex Loes) Sprague, KF282183; *E.myrianthus* Hemsl., KF282184, KF282185, KF282186; *E.nanoides* Loes. ex Rehder, KF282187; *E.nitidus* Benth., KF282188; *E.oblongifolius* Loes. ex Rehder, KF282189, KF282190; *E.oxyphyllus* Miq., KF282191, KF282192; *E.phellomana* Loes. ex Diels, KF282193, KF282194; *E.porphyreus* Loes., KF282195, KF282196; *E.sanguinea* Loes. ex Diels, KF282197, KF282198; *E.semenovii* Regel & Herder, KF282199; *E.sp.*, KF282200; *E.subsessilis* Sprague, KF282201, KF282202; *E.theacola* C. Y. Cheng ex T. L. Xu & Q. H. Chen, KF282203; *E.tingens* Wall., KF282204; *E.verrucosa* Scop., KF282205; *E.verrucosoides* Loes., KF282206; *E.viburnoides* Prain, KF282207; *E.wilsonii* Sprague, KF282208; *Celastrusorbiculatus* Thunb., KF282209; *Glyptopetalumcontinentale* (Chun & How) C. Y. Cheng & J. S. Ma, KF282210; *G.pallidifolium* (Hayata) Q. R. Liu & S. Y. Meng, KF282192; *G.rhytidophyllum* (Chun & How) C. Y. Cheng, KF282211; *Triperygiumregelii* Sprague & Takeda, KF282212; *T.wilfordii* Hook. f., KF282213.

^1^ The terms gathering, duplicate, and specimen follow the definitions of ICN (Turland et al. 2018).

## References

[B1] Anonymous (2013) The China Biodiversity Red List: Higher Plants. Published by Ministry of Ecological and Environmental of the People’s Republic of China & Chinese Academy of Sciences. http://www.mee.gov.cn/gkml/hbb/bgg/201309/W020130917614244055331.pdf

[B2] Baillon HE (1877) Histoire des Plantes, vol. 6. Librairie Hachette, Paris, 523 pp.

[B3] Chang CY (1988) Celastraceae. In: Chang CY (Ed.) Flora Sichuanica, vol.4. Science and Technological Publishing House of Sichuan, Chengdu, 247–346.

[B4] Cheng CY Kao TC Ma QS Ma JS Huang PH (1999) Celastraceae. In: Cheng CY Huang PH (Eds) Flora Reipublicae Popularis Sinicae vol.45(3). Science Press, Beijing, 1–218.

[B5] Chun WY How FC (1958) Contributions to the Flora of South China (I). Zhiwu Fenlei Xuebao 7(1): 1–90.

[B6] Clifford HT Bostock PD (2007) Etymological Dictionary of Grass. Springer, Berlin, 319 pp.

[B7] Franchet A (1886) Plantas Yunnanenses a cl. J.M. Delavay collectas enumeravit novasque describit. Bulletin de la Société Botanique de France 33(6): 358–467. 10.1080/00378941.1886.10828470

[B8] Harris JG Harris MW (2001) Plant Identification Terminology: An illustrated glossary. Spring Lake Publishing, Spring Lake, 206 pp.

[B9] Hayata B (1913) Icones Plantarum Formosanarum nec non et Contributiones ad Floram Formosanam, vol. 3. Bureau of Productive Industries, Taihoku, 222 pp. [35 pls]

[B10] Hou D (1963) Two additional Asiatic species of Glyptopetalum (Celastraceae). Blumea 12: 57–60.

[B11] Hou J (2012) Research Institute of West China Academy of Sciences. Central Party Literature Press, Beijing, 295 pp.

[B12] IPNI (2022) International Plant Names Index. https://www.ipni.org/

[B13] IUCN (2022) Guidelines for Using the IUCN Red List Categories and Criteria, prepared by the Standards and Petitions Committee, version 15. http://www.iucnredlist.org/documents/RedListGuidelines.pdf

[B14] Katoh K Standley DM (2013) MAFFT multiple sequence alignment software version 7: Improvements in performance and usability. Molecular Biology and Evolution 30(4): 772–780. 10.1093/molbev/mst010 23329690 PMC3603318

[B15] Kurz S (1875) Contributions towards a knowledge of the Burmese flora (Part II). Journal of the Asiatic Society of Bengal 44(2): 128–190.

[B16] Kurz S (1877) Forest Flora of British Burma, vol. 1. Office of the Superintendent of Government Printing, Calcutta, 549 pp. 10.5962/bhl.title.52413

[B17] Li YN Xie L Li JY Zhang ZX (2014) Phylogeny of *Euonymus* inferred from molecular and morphological data. Journal of Systematics and Evolution 52(2): 149–160. 10.1111/jse.12068

[B18] Linnaeus C (1753) Species Plantarum, vol. 1–2. Impensis Laurenti Salvii, Holmiae, 1200 pp.

[B19] Loesener T (1901–1902) Übersicht über die bis jetzt bekannten chinesischen Celastraceen. Botanische Jahrbücher für Systematik, Pflanzengeschichte und Pflanzengeographie 30: 446–474.

[B20] Ma JS Zhang ZX Liu QR Peng H Funston M (2008) Celastraceae. In: Wu ZY Raven PH Hong DY (Eds) Flora of China, vol.11. Science Press, Beijing & Missouri Botanical Garden Press, St. Louis, 439–492.

[B21] Meng SY Wang JL Liu QR (2011) On the identity of *Euonymuspallidifolia* (Celastraceae). Annales Botanici Fennici 48(2): 185–187. 10.5735/085.048.0216

[B22] Miller P (1754) The Gardeners Dictionary: containing the methods of cultivating and improving all sorts of trees, plants, and flowers, for……, vol. 1, 4^th^ edn. Printed for the Author, London, without pagination, 534 pp. 10.5962/bhl.title.79061

[B23] Minh BQ Nguyen MAT von-Haeseler A (2013) Ultrafast approximation for phylogenetic bootstrap. Molecular Biology and Evolution 30(5): 1188–1195. 10.1093/molbev/mst024 23418397 PMC3670741

[B24] Nakai T (1941) Subdivisions of the genus *Euonymus*. Shokubutsu Kenkyu Zasshi 17: 615–619.

[B25] Nguyen LT Schmidt HA Von-Haeseler A Minh BQ (2014) IQ-TREE: A fast and effective stochastic algorithm for estimating Maximum-Likelihood phylogenies. Molecular Biology and Evolution 32(1): 268–274. 10.1093/molbev/msu300 25371430 PMC4271533

[B26] Posada D (2008) jModelTest: Phylogenetic model averaging. Molecular Biology and Evolution 25(7): 1253–1256. 10.1093/molbev/msn083 18397919

[B27] Prain D (1891) Noviciae Indicae IV. Two additional species of *Glyptopetalum*. Journal of the Asiatic Society of Bengal 60(2): 206–210.

[B28] Ronquist F Huelsenbeck JP (2003) MrBayes 3: Bayesian phylogenetic inference under mixed models. Bioinformatics 19(12): 1572–1574. 10.1093/bioinformatics/btg180 12912839

[B29] Roxburgh W (1824) Flora Indica or descriptions of Indian plants, vol. 2. Printed at the Mission Press, Serampore, 588 pp.

[B30] Sargent CS (1911–1913) PlantaeWilsonianae. An enumeration of the woody plants collected in western China for the Arnold Arboretum of Harvard University during the years 1907, 1908, and 1910 by E. H. Wilson, vol. 1. The University Press, Cambridge, 611 pp. 10.5962/bhl.title.191

[B31] Schneider C Rasband W Eliceiri K (2012) NIH Image to ImageJ: 25 years of image analysis. Nature Methods 9(7): 671–675. 10.1038/nmeth.2089 22930834 PMC5554542

[B32] Simmons MP (2004) Celastraceae. In: Kubitzki K (Eds) The Families and Genera of Vascular Plants, vol.VI: Flowering Plants. Dicotyledons: Celastrales, Oxalidales, Rosales, Cornales, Ericales (volume edited by Kubitzki K). Springer-Verlag, Berlin, 29–64. 10.1007/978-3-662-07257-8_6

[B33] Simmons MP Mckenna MJ Bacon CD Yakobson K Cappa JJ Archer RH Ford AJ (2012) Phylogeny of Celastraceae tribe Euonymeae inferred from morphological characters and nuclear and plastid genes. Molecular Phylogenetics and Evolution 62(1): 9–20. 10.1016/j.ympev.2011.08.022 22001302

[B34] Thiers B (2022) Index Herbariorum: a global directory of public herbaria and associated staff. New York Botanical Garden’s Virtual Herbarium. http://sweetgum.nybg.org/science/ih

[B35] Thwaites GK (1856) Description of new genera and species of Ceylon plants. Hooker’s Journal of Botany and Kew Garden Miscellany 8: 266–271.

[B36] Turczaninow N (1858) Animadversiones ad secundam partem herbaria Turczaninowiani nunc Universitatis caesareae Charkowiensis. Bulletin de la Société Impériale des Naturalistes de Moscou 31: 379–476.

[B37] Turland NJ Wiersema JH Barrie FR Greuter W Hawksworth DL Herendeen PS Knapp S Kusber WH Li DZ Marhold K May TW McNeill J Monro AM Prado J Price MJ Smith GF (2018) International Code of Nomenclature for algae, fungi, and plants (Shenzhen Code) adopted by the Nineteenth International Botanical Congress Shenzhen, China, July 2017. Regnum Vegetabile 159. Koeltz Botanical Books, Glashütten. 10.12705/Code.2018

[B38] Wilson EH (1913) A Naturalist in Western China with Vasculum, Camera, and Gun, vol. 1. Methuen & Co. Ltd., London, 251 pp. [59 pls]

[B39] Wilson EH (1929) China: Mother of Gardens. The Stratford Company, Boston, 408 pp.

[B40] Yin KP Wang HY Zhong SX (2010) Tracing one Hundred Years of Change: Illustrating the Environmental Changes in Western China. Encyclopedia of China Publishing House, Beijing, 582 pp.

